# Different aspects of frailty and COVID-19: points to consider in the current pandemic and future ones

**DOI:** 10.1186/s12877-021-02316-5

**Published:** 2021-06-27

**Authors:** Hani Hussien, Andra Nastasa, Mugurel Apetrii, Ionut Nistor, Mirko Petrovic, Adrian Covic

**Affiliations:** 1 Dr C I Parhon University Hospital, Department of Nephrology, Iasi, Romania; 2 Department of Internal Medicine, Nephrology and Geriatrics, Grigore T Popa University of Medicine and Pharmacy, Faculty of Medicine, Bd Carol nr 50, Iasi, Romania; 3 Section of Geriatrics, Department of Internal Medicine and Pediatrics, Ghent University, Ghent, Belgium

**Keywords:** COVID-19, Microbiota, Inflamm-ageing, Immunosenescence, Frailty, Vaccination, SARS co-V2, CFS

## Abstract

**Background:**

Older adults at a higher risk of adverse outcomes and mortality if they get infected with Severe acute respiratory syndrome coronavirus 2 (SARS- CoV-2). These undesired outcomes are because ageing is associated with other conditions like multimorbidity, frailty and disability. This paper describes the impact of frailty on coronavirus disease 2019 (COVID-19) management and outcomes. We also try to point out the role of inflamm-ageing, immunosenescence and reduced microbiota diversity in developing a severe form of COVID-19 and a different response to COVID-19 vaccination among older frail adults. Additionally, we attempt to highlight the impact of frailty on intensive care unit (ICU) outcomes, and hence, the rationale behind using frailty as an exclusion criterion for critical care admission. Similarly, the importance of using a time-saving, validated, sensitive, and user-friendly tool for frailty screening in an acute setting as COVID-19 triage.

We performed a narrative review. Publications from 1990 to March 2021 were identified by searching the electronic databases MEDLINE, CINAHL and SCOPUS.

Based on this search, we have found that in older frail adults, many mechanisms contribute to the severity of COVID-19, particularly cytokine storm; those mechanisms include lower immunological capacity and status of ongoing chronic inflammation and reduced gut microbiota diversity.

Higher degrees of frailty were associated with poor outcomes and higher mortality rates during and after ICU admission. Also, the response to COVID-19 vaccination among frail older adults might differ from the general population regarding effectiveness and side effects.

Researches also had shown that there are many tools for identifying frailty in an acute setting that could be used in COVID-19 triage, and before ICU admission, the clinical frailty scale (CFS) was the most recommended tool.

**Conclusion:**

Older frail adults have a pre-existing immunopathological base that puts them at a higher risk of undesired outcomes and mortality due to COVID-19 and poor response to COVID-19 vaccination. Also, their admission in ICU should depend on their degree of frailty rather than their chronological age, which is better to be screened using the CFS.

**Supplementary Information:**

The online version contains supplementary material available at 10.1186/s12877-021-02316-5.

## Background

By the end of February 2021, the number of COVID-19 cases has exceeded one hundred million globally [1]. The older adults were the most affected population segment in terms of hospitalisation, poor outcomes and mortality due to COVID-19 in Europe [2, 3], the United Kingdom (UK) [4], United States (US) [5] and Canada [6]. The high risk of mortality and poor outcomes among older adults diagnosed with COVID-19 is a natural output of the high prevalence of comorbidities, weak immune system, and, most importantly, frailty in this unique population.

Frailty is defined as an age-related clinical disorder, usually with a decline in multiple organ systems’ physiological ability, characterised by a higher degree of vulnerability to what appears to be a minor stressor which exposes frail older adults at a higher risk of poor health outcomes, including dependence and disability [7].

Frailty is induced by an underlying mechanism independent of ageing but most likely to evolve and proceed with the ageing process; however, frailty is not a necessary element of ageing, and many adults reach advanced age without becoming frail [8].

During the current COVID-19 pandemic, frailty is important since it is a common clinical syndrome in older adults. In a recent meta-analysis, including 1,750,000 adults aged ≥50 years from 62 countries, the overall prevalence of frailty was 12% [9]. These figures are consistent with 15% as an estimated prevalence of frailty among Europeans aged ≥65 years [10]. Moreover, almost ¾ of frail persons has multimorbidity [11].

Although the existence of frailty or multimorbidity was not associated with increased risk of SARS- CoV-2 infection [12] yet, frail older adults are at higher of developing severe COVID-19 than pre-frail or non-frail older adults [13]. Indeed, the presence of frailty necessitates complex medical care demands, including ICU admission, notwithstanding the scarce resources of healthcare systems in the current setting of the SARS-CoV-2 pandemic.

This article reviews the current literature to determine the impact of frailty on older adults diagnosed with COVID-19. Also, we explain the causes and mutual mechanisms (inflamm-ageing, immunosenescence and reduced microbiota diversity) by which frail adults are more susceptible to a higher risk of developing a severe form of COVID-19, adverse outcomes, mortality and a different response to vaccination. Similarly, we attempt to highlight the importance of identifying frail older people using an efficient screening tool before their hospitalisation or ICU admission.

### Main text

We performed this narrative review to discuss the impact of frailty on older adults diagnosed with COVID-19. Also, to underline mutual mechanisms (inflamm-ageing, immunosenescence and reduced microbiota diversity), frail older adults are more susceptible to a higher risk of developing a severe form of COVID-19, adverse outcomes, mortality and a different response to vaccination. Similarly, we attempt to highlight the importance of identifying frail older people using an efficient screening tool before their hospitalisation or ICU admission.

A literature search was conducted up to March 2021, using the electronic databases MEDLINE, CINAHL, and SCOPUS to identify the original articles, review articles, and editorials that focused on the conceptual or theoretical aspects of frailty in older adults and frail adults diagnosed with COVID-19.

We have used the following terms: frailty in older adults, frailty in elderly, frailty in geriatrics, frailty and ageing, frailty mechanisms, inflamm-ageing, immunosenescence, frailty and SARS-CoV2, frailty screening, frailty assessment, frailty tools, frailty instruments, frailty and COVID-19, ICU in frail adults, vaccination in frailty, reduced microbiota diversity in frailty.

We also included studies of any design, quantitative or qualitative, and available data from official websites. We limited the search to articles published in the English language only between 1990 and 2021.

We have found 2543 papers after removing duplicates and did not match our search eligibility criteria. Out of these, 467 papers were considered after the title and abstract assessment. After a full-text review, the final relevant papers were 100 papers.

### Inflamm-ageing, immunosenescence and reduced microbiota diversity: an ominous trinity in frail older adults diagnosed with COVID-19.

#### Inflamm-ageing and immunosenescence

As previously explained, older adults are at higher risk of poor outcomes because of ageing-associated conditions as frailty, multi-comorbidities, and weak immunity. In general, compared with the young, older adults have a decrease in their immune systems’ capacity to cope with infection, which is mostly the result of the altered immune response to pathogens [14].. This impairment in the immune system, which is associated with ageing, is called immunosenescence.

Notably, and in a close link with COVID-19, it is well documented that the risk of complications and death from respiratory infections among seniors rises with immunosenescence and concomitant lung and heart health issues [15] and that frailty is associated with lower recovery rates and significant adverse outcomes in older adults with acute respiratory infections [16].

In old age, there is impaired crosstalk between the immune system’s innate and adaptive arms and an ongoing chronic inflammation known as inflamm-ageing, a common biological factor responsible for frailty and the onset of some diseases in older persons [17, 18]. Inflamm-ageing is characterised by Chronic Low-grade Inflammatory Phenotype (CLIP), which is associated with a concomitant progressive increase in pro-inflammatory markers, cytokines including interleukin (IL)-6, IL-1b, and tumor necrosis factor (TNF)- α [19, 20]. Moreover, these ongoing inflammatory processes may impair the host’s ability to identify pathogens as a harmful signal [17, 21]. By association, immunosenescence and inflamm-ageing would significantly impact outcome and survival among frail adults during pandemics.

In addition to CLIP, it is well established that there is an impairment of naive T cells in terms of numbers and function in older persons, which results in adaptive immunity dysfunction [17, 22]. Similar patterns of inflammation were detected in patients with severe COVID-19, where there is a state of hyper-inflammation [23], with an increase in the levels of interferon-γ, TNF-α, C-Reactive Protein (CRP) and cytokines, in particular, IL-10, IL-6, and IL-17, which correlates with a significant reduction in T cells population, and even the surviving T cells are functionally exhausted with impaired proliferation [24–26].

Previous studies on older adults have shown that elevated serum IL-6 and CRP levels are associated with a significant risk of developing frailty and mortality [27, 28]. Another aspect of high relevance is the immunological similarity between COVID-19 and frailty regarding Cluster of Differentiation (CD) levels. Studies on autopsies from persons who died from COVID-19 were positive for immunity cells, including CD4, CD8, CD20, and CD38 [29]. Interestingly, immunogerontological studies among frail patients have shown a chronic increase in the same CD types [30–34].

Moreover, it is well documented that a higher serum level of pro-inflammatory cytokines in COVID-19 patients is associated with poor outcomes. A plethora of studies has shown that the elevation of IL-2 and IL-6 is correlated with COVID-19 replication and disease severity and that patients requiring ICU admission had higher concentrations of cytokines than those who were not requiring ICU admission [35–39]. Also, a higher level of interleukins is an indicator of poor prognosis and high mortality rates in patients with severe COVID-19 [25, 40].

Presumably, in addition to weak immunity in frail older persons, they have a pre-existing chronic inflammatory status with higher pro-inflammatory markers, which put them at a higher risk of developing a severe form of COVID-19 and higher mortality rates.

#### Microbiota: a playmaker in frailty and COVID-19

In addition to inflamm-ageing and immunosenescence, microbiota diversity reduction contributes to the weak immune system among frail older adults. The pathogens interactions with the immune system happen in an environment that is influenced by its endogenous microbiota, owing to their high capacity to regulate many immunity aspects, including innate and adaptive immunity, locally and at distant sites, in particular in the intestine and lungs [41–43].

Previous research has shown that because of the increased plasma levels of the major pro-inflammatory cytokines, older people’s microbiota reveals a more substantial interindividual variability than that of younger adults; also, the reduced gut microbiota diversity is associated with increased frailty [44–46]. Unsurprisingly, the grade of frailty is a better indicator of changes in gut microbiota than chronological age [47].

Studies have recently shown an alteration in gut microbiota among COVID-19 patients [48] and that the faecal microbiota alteration is associated with the higher fecal level of SARS-CoV-2 and a severe form of COVID-19 [49]. Consistently, biopsies from deceased persons infected with COVID-19 have shown a change in lung microbiota diversity, especially in those aged ≥65 or with comorbidities [50].

**Fig. 1:**
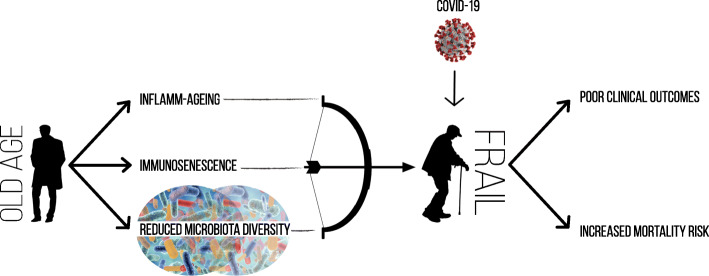
This figure shows the pathological mechanisms in older adults which expose frail patients with COVID-19 to undesired outcomes

Thus, among frail older adults, the susceptibility to infections, including SARS-CoV-2, depends on the interplay between immune capacity and body microbiota. Hence, the reduced microbiota diversity accompanied by immunosenescence and inflamm-ageing would predispose frail adults to develop a severe form of COVID-19. Therefore, understanding the role of microbiota in the pathogenesis of frailty and respiratory viral infections would allow for more targeted therapy for the frail population during the current pandemic and future ones. (See Fig. 1, Immunological factors contributing to developing a severe form of COVID-19 among frail older adults)

#### Frailty and COVID-19 vaccination

Another important aspect to consider in frail older adults is their potential response to the current COVID-19 vaccines. Although older adults are on the top of the COVID-19 vaccination list, frail ones were excluded from COVID-19 vaccines trials [51]. The inflamm-ageing and immunosenescence, which represent a cardinal element in the ageing process, are also associated with a poor immunological response to vaccination or previous infections [52], and this poor response would be worse among frail older adults [53, 54]. This known poor response to vaccination among frail older adults has triggered doctors, for example, in Norway, to assess for frailty before deciding whether to proceed with COVID-19 vaccination or not [55], particularly after recently 23 frail older adults have died shortly after receiving a COVID-19 vaccine [56].

Therefore, even after vaccination and because of their potential poor response to vaccines, it is predicted that older frail adults will be exposed to the same risk of infection or even, in the best case, a slightly lower risk than pre-vaccination. Hence, it is recommended to defer any early relaxation of the current community COVID-19 policies when dealing with this special population to maintain their protection.

### Critical care for frail older adults in COVID-19 pandemic: the battle of ventilators

As illustrated before, there is a refined relationship of frailty with poor outcomes in older adults infected with SARS-CoV-2, who also are burdened with other ageing-associated conditions, including the weak immune system, reduced gut microbiota, and comorbidities. This constellation of the ageing-associated conditions and frailty would attribute to COVID-19 severity, which will signal the need for ICU admission.

**Fig. 2:**
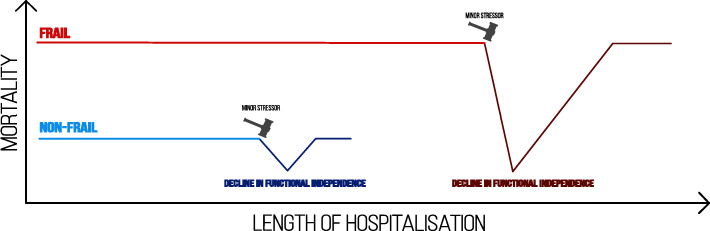
This figure shows that frailty in older adults is associated with prolonged hospitalisation, more significant decline in functional independence and higher mortality after exposure to minor stressors

In one recent study from 12 countries, which included five thousand hospitalised patients diagnosed with COVID 19 with a median age of 74, the degree of frailty was associated with high mortality rates and the necessity for a higher level of post-discharge care among survivors [57]. Consistently, the severe degree of frailty among COVID-19 patients was associated with prolonged hospitalisation, all-cause mortality, and higher mortality risk in the next 2 weeks following discharge [58–64]. (See Fig. 2, Frailty is associated with functional dependence, longer hospitalisation and higher mortality).

During the current pandemic, critical care services are overwhelmed, and there is a considerable shortage of ventilators worldwide. Thus, special arrangements should be taken to avoid disproportionate care, which is common in ICUs in Europe and North America [65]. Disproportionate care uses advanced life-sustaining measures in patients with poor long-term outcomes secondary to multiple chronic organ dysfunctions, comorbidities, and/or poor life quality [65]. Hence, the first goal is to take appropriate steps to optimise ICUs’ capacity by postponing non-emergency patient services and converting non-critical care units to critical care ones [66].

In the UK, the Intensive Care National Audit & Research Centre (ICNARC) has published a report on ICU admission data from England, Wales, and Northern Ireland, which shows that up to 31 August 2020, about 34% of patients admitted in ICU due to COVID-19 have died and that 66% death cases were in those who were ≥ 60 years [67]. Also, ICNARC has reported that from September 2020, 35.6% of patients admitted in ICU due to COVID-19 were aged ≥60 years and that 70% of invasively ventilated patients aged ≥70 have died in ICU. These figures confirm that older adults are at higher risk of being admitted to ICU due to COVID-19 and that they are more likely to die if they were invasively ventilated.

More than 50% of ICU admissions in the USA because of COVID-19, and over 80% of deaths were among adults aged ≥65 years [68]. Similar data were reported from Mainland China, with 80% of deaths among adults aged ≥60 years [68]. In a study that included 5700 patients hospitalised with COVID-19 in the New York City area, the mortality rate among patients aged ≥65 years who received mechanical ventilation was 97% compared to 76.4% aged 18–65 years [69].

Thus, a controversy had arisen over whether the old patients with COVID-19 should be admitted to ICU or whether they should be directed to palliative care management. Currently, there are two opposite strategies for approaching older adults, aiming to allocate mechanical ventilators. On the one hand, chronological age alone was used as exclusion criteria; for instance, doctors in Italy have opted for a cut-off of 65 years old in the case of pre-existing comorbidities [70]. Similarly, in Switzerland, the Swiss Academy of Medical Sciences (SAMS) [71] has published new guidelines for admission in ICU, stating that in the context of COVID-19, age is a risk factor for mortality and should be taken into consideration, yet without specifying a cut-off. SAMS recommends, “For ICU admission, the highest priority is to be accorded to those patients whose prognosis with regard to hospital discharge is good with intensive care, but poor without it” [71].

While, in the USA, the New York state department of health has chosen “saving the most lives” guidance, which allocates ventilators according to the presence of specific exclusion criteria and a cut-off of Sequential Organ Failure Assessment (SOFA) score [72]. In Spain, the ministry of health has announced on 03 April 2020, general criteria for ICU admission; the first criterion was “Non-discrimination for any reason beyond the patient’s clinical situation and their objective, evidence-based expectations of survival “, which makes the patient’s age out of the picture [73].

However, other stratifying ways have been used; for instance, in Pennsylvania, USA, allocating ventilators depends on calculating a specific score, including age and multi-comorbidities [74]. In the UK, the National Institute for Health and Care Excellence (NICE) has updated its guideline on critical care on 25 March 2020 to involve frailty screening for all older adults who present in COVID-19 triage irrespective of their COVID-19 status [75].

Meanwhile, in Switzerland, the Board of the Association for Geriatric Palliative Medicine (FGPG) has recommended Advance Care Planning (ACP) when managing older frail adults diagnosed with COVID-19 [76]. ACP allows frail older adults to opt for either hospitalisation or palliative care before infection or at least at the time of diagnosis, which respects the patient’s wishes, and hence, is ethically accepted.

Previous researches have shown that persons dying at an older age generally have more disability, but not a disease, than those dying at a younger age, and that a large proportion of their total years spent in the disabled state will contribute to the years just before their end-of-life [77].

Nevertheless, once frailty overlaps with comorbidities or disability, this is the moment of no return, and frailty will be a pre-death phase. Consistently, the short-term survival after admission in ICU of older adults has inversely associated with the degree of frailty in advanced age [78]; also, pre-ICU frailty correlates with a higher post-ICU disability and new admission in nursing homes among ICU survivors [79]. Accordingly, and due to escalating needs to allocate ventilators to those more likely to benefit and avoid mechanical ventilation withdrawal, physicians should proactively participate in conversations with patients and caregivers concerning do-not-intubate orders for high-risk subgroups of patients before their health deteriorates [80].

The COVID-19 in Older PEople (COPE) study, which included 1564 non-ICU patients diagnosed with COVID-19 (median age of 74 years), has shown that COVID-19 outcomes were better predicted by the degree of frailty than either chronological age or comorbidity [59]. Similarly, severe frailty is an independent predictor for mechanical ventilation among older adults diagnosed with COVID-19 [61]. Therefore, frailty screening among older adults before ICU admission is of central importance since it can guide clinicians to the ICU outcome (See Fig. 2).

Given the connection between frailty and fewer chances to be home discharged, and the development of adverse outcomes in the acute care setting, it seems fair to assume that a COVID-19 older adult with a high degree of frailty or disability is relatively closer to death than non-frail patients of the same age and are less likely to benefit from the critical care service.

Hence, it is clear that the potential profit of the admission of an old patient positive with COVID − 19 in critical care service cannot be rationally taken without assessing their frailty state before ICU admission. Frailty as a selection criterion for ICU admission is expected to deliver a more accurate, rational, yet ethically accepted choice during the time of pandemics. Ultimately, despite the debate on old patients with COVID-19, if they should receive treatment in ICU or not, there is no disagreement on the catastrophic impact of COVID-19 on old individuals admitted in the ICU, their families, and society as a whole.

### Frailty screening in COVID-19 triage

In general, all older adults should be assessed for frailty when encountered with healthcare staff because frailty is a complex condition that necessitates a particular intervention, yet an individualised one. Ideally, in case of emergencies, ambulance staff should recognise frail patients in the community because it would decrease the number of older adults attending the busy emergency department (ED). Nevertheless, it is not always applicable, in particular during times of pandemics.

The aim of screening for frailty in ED is to understand the acute manifestations of the present illness regarding the pre-existing health condition to predict adverse outcomes during hospitalisation or after discharge and prevent potential adverse outcomes [81]. Also, frailty screening guarantees more informed medical decision-making for both patients regarding treatment preferences and physicians in terms of triaging and therapy suitability [82]. Moreover, the outcome of frailty assessment in ED will further trigger the clinician’s decision and the patient and his family, and some patients might be admitted to the hospital, while others might opt for palliative care in the home. Even in the case of hospital admission, the management will be different at a detailed level. For example, deprescribing some of the current medications, avoiding some new drugs or manoeuvres and subsequently a different individualised approach in managing the current illness, because some interventions might be clinically less efficient, not only that but maybe more harmful.

There is a wide variety of frailty screening instruments, each with a range of included components. In a systematic review that includes 96 studies, 51 frailty tools were identified for screening and diagnosis of frailty in outpatient (OPD) and inpatient (IPD) departments [83]. (see supplementary Table 1).

However, besides simplicity and sensitivity, an optimal screening tool must be efficient in countries with scarce resources. Indeed, most of the instruments for frailty assessment are too complicated for use in acute care situations. Some more straightforward tools involve a type of manual evaluation approach that may be time-consuming, prone to inter-operator error and might expose the assessor to further infection risk. Nevertheless, recent research, including three hundred thousand adults, has shown that frailty is associated with more severe COVID-19 and higher mortality rates regardless of the assessment tool used [84].

A well-validated frailty tool that includes physical assessment is the frailty phenotype score which requires patients to perform physical maneuvers, such as handgrip strength and gait-speed assessments [85], that are hard to assess during pandemics. Even non-physical tools could be too long to be used in the triage of pandemics, for example, the Edmonton Frail Scale (EFS) [86] and the Groningen Frailty Indicator (GFI) [87], yet the former was used for assessment of frailty among older adults diagnosed with COVID-19 [88].

The Hospital Frailty Risk Score (HFRS) developed by Thomas Gilbert and colleagues [89] is another validated, systemic, and low-cost tool to identify hospitalised frail people at risk for mortality and adverse outcomes. It generates electronic health record data, and it has the advantage that it can be calculated instantly upon or just before admission. The HFRS was efficiently used for frailty screening in eighteen thousand older adults (≥ 65 years) diagnosed with COVID-19 [90]; however, one study including 4000 adults admitted to ICU has shown that HFRS did not independently predict the outcome of ICU patients ≥75 years [91]. Nevertheless, the HRFS needs electronic health records to be available in a *nationwide health* information network that contains the ICD-10 diagnostic codes of all the previous inpatient and outpatient admissions, which in some limited-resources health systems is difficult, if not impossible. Similarly, the Frailty Index (FI) is a well-validated tool for frailty screening in the general population [92], and it was used with COVID-19 patients [93]. However, the FI requires laboratory tests and some previous medical records, which might not be available in many health facilities.

Other short tools recommended for frailty screening in the emergency department include PRISMA-7 [94, 95], FRAIL scale [96], and The Clinical Frailty Scale (CFS) [97]. PRISMA-7 defines an older adult as frail or non-frail without referring to the level of frailty. The FRAIL scale is a self-reporting test [96], and it was used for frailty assessment among older adults diagnosed with COVID-19 [13]. (see supplementary Table 2).

The CFS depends on both the assessor’s clinical judgment and information from prior geriatric assessments. It classifies frailty in old persons to nine grades, where grade 1 is very fit, and grade 9 is terminally ill. The CFS considers the cognitive function, mobility, comorbidities, and functional status combined into a pictograph [97].

Predictably, the CFS was recommended for frailty screening in the emergency setting and by critical care staff, owing to its excellent predictability of mortality and the length of hospitalization, not only that, but also it was the most comprehensive user-friendly tool, yet less exigent [94, 98–100]. Similarly, the European Very elderly Intensive Patient (VIP2) study, which included 4000 older adults (≥ 80 years) admitted to the ICU, has shown that the CFS score was inversely associated with short-term survival [101].

One of CFS’s most significant advantages is that it does not require any physical evaluation that is challenging to be performed in the ED. Also, it easy to use in multiple settings, including the acute general medical setting, even by non-trained junior doctors [102, 103]. This Feasibility of CFS gives it ancillary benefits in health systems with limited means where there is a lack of experienced doctors, as well as in pandemics time when rapid decisions are required in busy ED Recently, the International Conference of Frailty and Sarcopenia Research (ICFSR) has recommended in their 2019 guidelines, the CFS, as a screening tool for frailty [104].

During the current pandemic, the National Institute for Health and Care Excellence (NICE) has opted for using the CFS as a screening tool in COVID-19 triage. NICE has chosen the degree of frailty in older adults as a filtering criterion for hospitalisation and critical care admission, where the assessor should ask for a patient’s capability 2 weeks ago before their presentation in ED. NICE also recommends that a COVID positive patient with CFS ≥ 5 be managed initially outside critical care [75]. Similarly, Nederland guidelines [105] and the Belgian Society of Intensive care medicine have recommended using the CFS in COVID-19 triage [66].

Therefore, during the current pandemic, many studies have used CFS to screen frailty among older adults diagnosed with COVID-19 [59–61, 105–109]. Indeed, many studies have shown that higher CFS scores were associated with prolonged hospitalisation, poor outcomes, and higher mortality rates among older adults diagnosed with COVID-19 [59, 105, 109].

It should be noticed that a CFS cut-off ≥5 is not absolute, while it was recommended by NICE [75], studies from France [109], the COMET study from 11 European countries has shown that CFS cut-off ≥6 was a more suitable risk marker for mortality among frail adults (≥ 65 years old) diagnosed with COVID-19 [110]. However, a study from Australia and New Zealand that includes 10,000 adults (median age 64 years) has shown that a CFS score of less than 7 was not strongly associated with mortality [63].

**Fig. 3:**
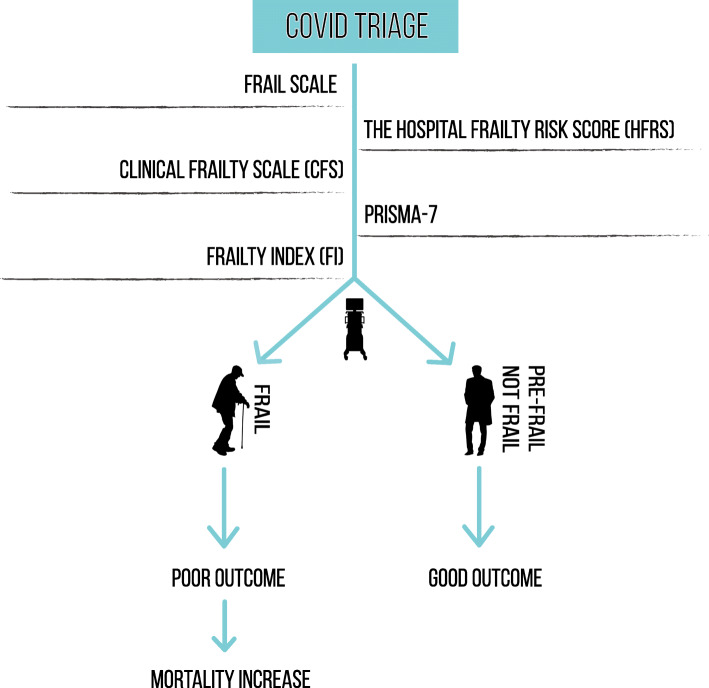
This figure shows that the allocation of ventilators to non-frail and pre-frail older adults was associated with better outcomes and lower mortality rates

Nevertheless, it is crucial to understand that frailty does not define futility in older adults and that the decisions to hospitalise a patient or admit them to ICU are beset with difficulties. Such difficulties are because, to the best of our knowledge, there is no yet a studied cut-off of any frailty instrument, which defines the patient who would benefit from ICU admission. Indeed, the frontier between “futility” and “worthiness” of ICU admission for old frail individuals is faint. (See Fig. 3, Frailty screening and allocation of ventilators in ICU).

Owing to its feasibility and accuracy, the CFS is hence proposed to be a handy tool in identifying frailty, predicting the length of stay, and mortality among old adults who present in emergency settings and presumably, in the triage of COVID-19. Finally, categorising older adults according to their frailty degree will permit electing those requiting full therapeutic options and those who should be managed in the palliative care setting, like nursing homes, without admitting them to the hospital. Therefore, new guidelines should be constructed that address the management of frail old adults in times of pandemics, mainly when there is a surge in health care demands, without compromising both ethical and clinical aspects.

## Conclusions

The frail old population is a special segment of the population with their particularities, distinguishing them from the rest. Older adults, in particular, frail ones, have a weaker immune system, reduced gut microbiota diversity, and longstanding inflammatory status than the general population. Those factors contribute to the severity of COVID-19 and the high mortality rate. Moreover, frailty in patients with COVOD-19 is associated with poor outcomes, mortality in ICU, re-admission and short survival post-ICU discharge. Moreover, frailty is associated with a poor response to vaccination and more side effects, and hence, as a precautionary measure, it might be reasonable to screen older adults for frailty before vaccination.

The allocating of healthcare resources, mainly mechanical ventilators, is a wise requisite in times of pandemics. Therefore, we suggest that the decision of “NO ICU “admission for older persons should depend on their degree of frailty as a primary selection criterion and that excluding patients based merely on their age is unreliable. The assessment of frailty in COVID-19 or any pandemic triage is thus mandatory to define priorities and actions, and it would provide essential information to evaluate the efficiency of COVID-19 management. Owing to its feasibility, user-friendliness and sensitivity, together with the prediction of poor outcomes and mortality among COVID-19 frail adults, we suggest that the Clinical frailty scale is the screening tool of choice in COVID-19 triage.

Our findings imply that the optimisation of treatment and management of older adults in COVID-19 and future pandemics may differ between frail and non-frail individuals, and hence, cannot be achieved without proper frailty assessment before hospital admission. Accordingly, several policy implications should be considered in dealing with frail old adults in pandemics; additional research is required to delineate more clearly the role of frailty in pandemics as cause and effect.

## Supplementary Information

**Additional file 1: Supplementary Table 1.** The main frailty screening tools used in clinical practice.

**Additional file 2: Table 2.** Frailty assessment tools that were used during the COVOD-19 pandemic.

## Data Availability

Not applicable.

## Abbreviations

ACP: Advance Care Planning
CFS: Clinical frailty scale
CLIP: Chronic Low-grade Inflammatory Phenotype
CD: Cluster of Differentiation
COPE: COVID-19 in Older PEople
COVID-19: Coronavirus disease 2019
CRP: C-reactive Protein
ED: Emergency department.
HFRS: The Hospital Frailty Risk Score
ICFSR: International Conference of Frailty and Sarcopenia Research
ICNARC: Intensive Care National Audit & Research Centre
ICU: Intensive care unit
IFN: Interferon
IL: Interleukin
IPD: Inpatient department
NICE: The National Institute for Health and Care Excellence
OPD: Outpatient department
SAMS: Swiss Academy of Medical Sciences
SARS-CoV-2: Severe acute respiratory syndrome coronavirus 2
SOFA: Sequential Organ Failure Assessment
TNF: Tumor Necrosis Factor

## References

[1] JHU. How does mortality differ across countries? : John Hopkins Coronavirus resource Centre 2020 [Available from: https://coronavirus.jhu.edu/data/mortality. [accessed 2020 July 26].

[2] ESSy. Surveillance of COVID-19 at longterm care facilities in the EU/EEA. 2020 [Available from: https://www.ecdc.europa.eu/sites/default/files/documents/covid-19-long-term-care-facilities-surveillance-guidance.pdf. [accessed 2022 July 22].

[3] Cohen JF, Korevaar DA, Matczak S, Brice J, Chalumeau M, Toubiana J. COVID-19-related mortality by age groups in Europe: A meta-analysis. medRxiv. 2020. 10.3389/fmed.2020.560685 PMC7840596 33521004

[4] ONS. Deaths involving coronavirus (COVID-19) UK: Office for national statistics; 2021 [cited 2021 February 2021]. Available from: https://www.ons.gov.uk/peoplepopulationandcommunity/healthandsocialcare/conditionsanddiseases/articles/coronaviruscovid19/latestinsights#deaths. [accessed 2021 21.02.2021].

[5] CDC. Provisional COVID-19 Death Counts by Age. 2021 [cited 2021 February 21]. Available from: https://data.cdc.gov/NCHS/Provisional-COVID-19-Death-Counts-by-Sex-Age-and-W/vsak-wrfu/data. [].

[6] HealthInfobase. Coronavirus disease 2019 (COVID-19): Epidemiology update. 2021 [cited 2021 February 21]. Available from: https://health-infobase.canada.ca/covid-19/epidemiological-summary-covid-19-cases.html. [].

[7] Dent E, Martin FC, Bergman H, Woo J, Romero-Ortuno R, Walston JD (2019). Management of frailty: opportunities, challenges, and future directions. Lancet..

[8] Clegg A, Young J, Iliffe S, Rikkert MO, Rockwood K (2013). Frailty in elderly people. Lancet..

[9] O'Caoimh R, Sezgin D, O'Donovan MR, Molloy DW, Clegg A, Rockwood K (2021). Prevalence of frailty in 62 countries across the world: a systematic review and meta-analysis of population-level studies. Age Ageing.

[10] O'Caoimh R, Galluzzo L, Rodriguez-Laso A, Van der Heyden J, Ranhoff AH, Lamprini-Koula M (2018). Prevalence of frailty at population level in European ADVANTAGE joint action member states: a systematic review and meta-analysis. Ann Ist Super Sanita.

[11] Vetrano DL, Palmer K, Marengoni A, Marzetti E, Lattanzio F, Roller-Wirnsberger R, Lopez Samaniego L, Rodríguez-Mañas L, Bernabei R, onder G, Joint Action ADVANTAGE WP4 Group (2019). Frailty and multimorbidity: a systematic review and meta-analysis. J Gerontol A Biol Sci Med Sci.

[12] Woolford SJ, D'Angelo S, Curtis EM, Parsons CM, Ward KA, Dennison EM (2020). COVID-19 and associations with frailty and multimorbidity: a prospective analysis of UK biobank participants. Aging Clin Exp Res.

[13] Ma Y, Hou L, Yang X, Huang Z, Yang X, Zhao N, He M, Shi Y, Kang Y, Yue J, Wu C (2020). The association between frailty and severe disease among COVID-19 patients aged over 60 years in China: a prospective cohort study. BMC Med.

[14] Linton PJ, Dorshkind K (2004). Age-related changes in lymphocyte development and function. Nat Immunol.

[15] Gruver AL, Hudson LL, Sempowski GD (2007). Immunosenescence of ageing. J Pathol.

[16] Lees C, Godin J, McElhaney JE, McNeil SA, Loeb M, Hatchette TF (2020). Frailty hinders recovery from influenza and acute respiratory illness in older adults. J Infect Dis.

[17] Weinberger B, Herndler-Brandstetter D, Schwanninger A, Weiskopf D, Grubeck-Loebenstein B (2008). Biology of immune responses to vaccines in elderly persons. Clin Infect Dis.

[18] Cevenini E, Monti D, Franceschi C (2013). Inflamm-ageing. Curr Opin Clin Nutr Metab Care.

[19] Franceschi C, Bonafe M, Valensin S, Olivieri F, De Luca M, Ottaviani E (2000). Inflamm-aging. An evolutionary perspective on immunosenescence. Ann N Y Acad Sci.

[20] Krabbe KS, Pedersen M, Bruunsgaard H (2004). Inflammatory mediators in the elderly. Exp Gerontol.

[21] Chen Y, Liu S, Leng SX (2019). Chronic low-grade inflammatory phenotype (CLIP) and senescent immune dysregulation. Clin Ther.

[22] Fagnoni FF, Vescovini R, Passeri G, Bologna G, Pedrazzoni M, Lavagetto G, Casti A, Franceschi C, Passeri M, Sansoni P (2000). Shortage of circulating naive CD8(+) T cells provides new insights on immunodeficiency in aging. Blood..

[23] Merad M, Martin JC. Pathological inflammation in patients with COVID-19: a key role for monocytes and macrophages. Nat Rev Immunol. 2020:1–8. 10.1038/s41577-020-0331-4 PMC7201395 32376901

[24] Diao B, Wang C, Tan Y, Chen X, Liu Y, Ning L, Chen L, Li M, Liu Y, Wang G, Yuan Z, Feng Z, Zhang Y, Wu Y, Chen Y (2020). Reduction and functional exhaustion of T cells in patients with coronavirus disease 2019 (COVID-19). Front Immunol.

[25] Wu C, Chen X, Cai Y, Xia J, Zhou X, Xu S (2020). Risk factors associated with acute respiratory distress syndrome and death in patients with coronavirus disease 2019 pneumonia in Wuhan. China JAMA Intern Med.

[26] De Biasi S, Meschiari M, Gibellini L, Bellinazzi C, Borella R, Fidanza L (2020). Marked T cell activation, senescence, exhaustion and skewing towards TH17 in patients with COVID-19 pneumonia. Nat Commun.

[27] Giovannini S, Onder G, Liperoti R, Russo A, Carter C, Capoluongo E (2011). Interleukin-6, C-reactive protein, and tumor necrosis factor-alpha as predictors of mortality in frail, community-living elderly individuals. J Am Geriatr Soc.

[28] Langmann GA, Perera S, Ferchak MA, Nace DA, Resnick NM, Greenspan SL (2017). Inflammatory markers and frailty in long-term care residents. J Am Geriatr Soc.

[29] Luo WY, H.; Gou, J.; Li, X.; Sun, Y.; Li, J.; Liu, L. Clinical Pathology of Critical Patient with Novel Coronavirus Pneumonia (COVID-19). . Preprints 2020, 2020020407. 2020.

[30] Leng SX, Margolick JB (2015). Understanding frailty, aging, and inflammation in HIV infection. Curr HIV/AIDS Rep.

[31] De Fanis U, Wang GC, Fedarko NS, Walston JD, Casolaro V, Leng SX (2008). T-lymphocytes expressing CC chemokine receptor-5 are increased in frail older adults. J Am Geriatr Soc.

[32] Lu Y, Tan CT, Nyunt MS, Mok EW, Camous X, Kared H, et al. Inflammatory and immune markers associated with physical frailty syndrome: findings from Singapore longitudinal aging studies. Oncotarget. 2016;7(20):28783–95. DOI: 10.18632/oncotarget.8939. 10.18632/oncotarget.8939 PMC5045356 27119508

[33] Ng TP, Camous X, Nyunt MSZ, Vasudev A, Tan CTY, Feng L, Fulop T, Yap KB, Larbi A (2015). Markers of T-cell senescence and physical frailty: insights from Singapore longitudinal ageing studies. NPJ Aging Mech Dis.

[34] Semba RD, Margolick JB, Leng S, Walston J, Ricks MO, Fried LP (2005). T cell subsets and mortality in older community-dwelling women. Exp Gerontol.

[35] Shi Y, Tan M, Chen X, Liu Y, Huang J, Ou J, et al. Immunopathological characteristics of coronavirus disease 2019 cases in Guangzhou, China. medRxiv. 2020:2020.03.12.20034736. DOI: 10.1101/2020.03.12.20034736. 10.1111/imm.13223 PMC7283723 32460357

[36] Yuan J, Zou R, Zeng L, Kou S, Lan J, Li X, et al. The correlation between viral clearance and biochemical outcomes of 94 COVID-19 infected discharged patients. Inflamm Res. 2020:1–8. 10.1007/s00011-020-01342-0 PMC7103893 32227274

[37] Gao Y, Li T, Han M, Li X, Wu D, Xu Y, Zhu Y, Liu Y, Wang X, Wang L (2020). Diagnostic utility of clinical laboratory data determinations for patients with the severe COVID-19. J Med Virol.

[38] Conti P, Ronconi G, Caraffa A, Gallenga CE, Ross R, Frydas I, et al. Induction of pro-inflammatory cytokines (IL-1 and IL-6) and lung inflammation by Coronavirus-19 (COVI-19 or SARS-CoV-2): anti-inflammatory strategies. J Biol Regul Homeost Agents. 2020;34(2):327–31. DOI: 10.23812/CONTI-E. 10.23812/CONTI-E 32171193

[39] Huang C, Wang Y, Li X, Ren L, Zhao J, Hu Y, Zhang L, Fan G, Xu J, Gu X, Cheng Z, Yu T, Xia J, Wei Y, Wu W, Xie X, Yin W, Li H, Liu M, Xiao Y, Gao H, Guo L, Xie J, Wang G, Jiang R, Gao Z, Jin Q, Wang J, Cao B (2020). Clinical features of patients infected with 2019 novel coronavirus in Wuhan, China. Lancet.

[40] Cao X (2020). COVID-19: immunopathology and its implications for therapy. Nat Rev Immunol..

[41] Belkaid Y, Harrison OJ (2017). Homeostatic immunity and the microbiota. Immunity..

[42] Hooper LV, Macpherson AJ (2010). Immune adaptations that maintain homeostasis with the intestinal microbiota. Nat Rev Immunol.

[43] Maschirow L, Suttorp N, Opitz B (2019). Microbiota-dependent regulation of antimicrobial immunity in the lung. Am J Respir Cell Mol Biol.

[44] Claesson MJ, Jeffery IB, Conde S, Power SE, O'Connor EM, Cusack S (2012). Gut microbiota composition correlates with diet and health in the elderly. Nature..

[45] Jackson MA, Jeffery IB, Beaumont M, Bell JT, Clark AG, Ley RE, O’Toole PW, Spector TD, Steves CJ (2016). Signatures of early frailty in the gut microbiota. Genome Med.

[46] Biagi E, Nylund L, Candela M, Ostan R, Bucci L, Pini E, Nikkïla J, Monti D, Satokari R, Franceschi C, Brigidi P, de Vos W (2010). Through ageing, and beyond: gut microbiota and inflammatory status in seniors and centenarians. PLoS One.

[47] Maffei VJ, Kim S, Blanchard E, Luo M, Jazwinski SM, Taylor CM (2017). Biological aging and the human gut microbiota. J Gerontol A Biol Sci Med Sci.

[48] Gu S, Chen Y, Wu Z, Chen Y, Gao H, Lv L, Guo F, Zhang X, Luo R, Huang C, Lu H, Zheng B, Zhang J, Yan R, Zhang H, Jiang H, Xu Q, Guo J, Gong Y, Tang L, Li L (2020). Alterations of the gut microbiota in patients with COVID-19 or H1N1 influenza. Clin Infect Dis.

[49] Zuo T, Zhang F, Lui GCY, Yeoh YK, Li AYL, Zhan H, Wan Y, Chung ACK, Cheung CP, Chen N, Lai CKC, Chen Z, Tso EYK, Fung KSC, Chan V, Ling L, Joynt G, Hui DSC, Chan FKL, Chan PKS, Ng SC (2020). Alterations in gut microbiota of patients with COVID-19 during time of hospitalization. Gastroenterology..

[50] Fan J, Li X, Gao Y, Zhou J, Wang S, Huang B, Wu J, Cao Q, Chen Y, Wang Z, Luo D, Zhou T, Li R, Shang Y, Nie X (2020). The lung tissue microbiota features of 20 deceased patients with COVID-19. J Inf Secur.

[51] Ramasamy MN, Minassian AM, Ewer KJ, Flaxman AL, Folegatti PM, Owens DR, Voysey M, Aley PK, Angus B, Babbage G, Belij-Rammerstorfer S, Berry L, Bibi S, Bittaye M, Cathie K, Chappell H, Charlton S, Cicconi P, Clutterbuck EA, Colin-Jones R, Dold C, Emary KRW, Fedosyuk S, Fuskova M, Gbesemete D, Green C, Hallis B, Hou MM, Jenkin D, Joe CCD, Kelly EJ, Kerridge S, Lawrie AM, Lelliott A, Lwin MN, Makinson R, Marchevsky NG, Mujadidi Y, Munro APS, Pacurar M, Plested E, Rand J, Rawlinson T, Rhead S, Robinson H, Ritchie AJ, Ross-Russell AL, Saich S, Singh N, Smith CC, Snape MD, Song R, Tarrant R, Themistocleous Y, Thomas KM, Villafana TL, Warren SC, Watson MEE, Douglas AD, Hill AVS, Lambe T, Gilbert SC, Faust SN, Pollard AJ, Aboagye J, Adams K, Ali A, Allen ER, Allen L, Allison JL, Andritsou F, Anslow R, Arbe-Barnes EH, Baker M, Baker N, Baker P, Baleanu I, Barker D, Barnes E, Barrett JR, Barrett K, Bates L, Batten A, Beadon K, Beckley R, Bellamy D, Berg A, Bermejo L, Berrie E, Beveridge A, Bewley K, Bijker EM, Birch G, Blackwell L, Bletchly H, Blundell CL, Blundell SR, Bolam E, Boland E, Bormans D, Borthwick N, Boukas K, Bower T, Bowring F, Boyd A, Brenner T, Brown P, Brown-O'Sullivan C, Bruce S, Brunt E, Burbage J, Burgoyne J, Buttigieg KR, Byard N, Cabera Puig I, Camara S, Cao M, Cappuccini F, Carr M, Carroll MW, Cashen P, Cavey A, Chadwick J, Challis R, Chapman D, Charles D, Chelysheva I, Cho JS, Cifuentes L, Clark E, Collins S, Conlon CP, Coombes NS, Cooper R, Cooper C, Crocker WEM, Crosbie S, Cullen D, Cunningham C, Cuthbertson F, Datoo BE, Dando L, Datoo MS, Datta C, Davies H, Davies S, Davis EJ, Davis J, Dearlove D, Demissie T, di Marco S, di Maso C, DiTirro D, Docksey C, Dong T, Donnellan FR, Douglas N, Downing C, Drake J, Drake-Brockman R, Drury RE, Dunachie SJ, Edwards CJ, Edwards NJ, el Muhanna O, Elias SC, Elliott RS, Elmore MJ, English MR, Felle S, Feng S, Ferreira da Silva C, Field S, Fisher R, Fixmer C, Ford KJ, Fowler J, Francis E, Frater J, Furze J, Galian-Rubio P, Galloway C, Garlant H, Gavrila M, Gibbons F, Gibbons K, Gilbride C, Gill H, Godwin K, Gordon-Quayle K, Gorini G, Goulston L, Grabau C, Gracie L, Graham N, Greenwood N, Griffiths O, Gupta G, Hamilton E, Hanumunthadu B, Harris SA, Harris T, Harrison D, Hart TC, Hartnell B, Haskell L, Hawkins S, Henry JA, Hermosin Herrera M, Hill D, Hill J, Hodges G, Hodgson SHC, Horton KL, Howe E, Howell N, Howes J, Huang B, Humphreys J, Humphries HE, Iveson P, Jackson F, Jackson S, Jauregui S, Jeffers H, Jones B, Jones CE, Jones E, Jones K, Joshi A, Kailath R, Keen J, Kelly DM, Kelly S, Kelly D, Kerr D, Khan L, Khozoee B, Killen A, Kinch J, King LDW, King TB, Kingham L, Klenerman P, Knight JC, Knott D, Koleva S, Lang G, Larkworthy CW, Larwood JPJ, Law R, Lee A, Lee KYN, Lees EA, Leung S, Li Y, Lias AM, Linder A, Lipworth S, Liu S, Liu X, Lloyd S, Loew L, Lopez Ramon R, Madhavan M, Mainwaring DO, Mallett G, Mansatta K, Marinou S, Marius P, Marlow E, Marriott P, Marshall JL, Martin J, Masters S, McEwan J, McGlashan JL, McInroy L, McRobert N, Megson C, Mentzer AJ, Mirtorabi N, Mitton C, Moore M, Moran M, Morey E, Morgans R, Morris SJ, Morrison HM, Morshead G, Morter R, Moya NA, Mukhopadhyay E, Muller J, Munro C, Murphy S, Mweu P, Noé A, Nugent FL, O'Brien K, O'Connor D, Oguti B, Olchawski V, Oliveira C, O'Reilly PJ, Osborne P, Owen L, Owino N, Papageorgiou P, Parracho H, Parsons K, Patel B, Patrick-Smith M, Peng Y, Penn EJ, Peralta-Alvarez MP, Perring J, Petropoulos C, Phillips DJ, Pipini D, Pollard S, Poulton I, Pratt D, Presland L, Proud PC, Provstgaard-Morys S, Pueschel S, Pulido D, Rabara R, Radia K, Rajapaska D, Ramos Lopez F, Ratcliffe H, Rayhan S, Rees B, Reyes Pabon E, Roberts H, Robertson I, Roche S, Rollier CS, Romani R, Rose Z, Rudiansyah I, Sabheha S, Salvador S, Sanders H, Sanders K, Satti I, Sayce C, Schmid AB, Schofield E, Screaton G, Sedik C, Seddiqi S, Segireddy RR, Selby B, Shaik I, Sharpe HR, Shaw R, Shea A, Silk S, Silva-Reyes L, Skelly DT, Smith DJ, Smith DC, Smith N, Spencer AJ, Spoors L, Stafford E, Stamford I, Stockdale L, Stockley D, Stockwell LV, Stokes M, Strickland LH, Stuart A, Sulaiman S, Summerton E, Swash Z, Szigeti A, Tahiri-Alaoui A, Tanner R, Taylor I, Taylor K, Taylor U, te Water Naude R, Themistocleous A, Thomas M, Thomas TM, Thompson A, Thompson K, Thornton-Jones V, Tinh L, Tomic A, Tonks S, Towner J, Tran N, Tree JA, Truby A, Turner C, Turner R, Ulaszewska M, Varughese R, Verbart D, Verheul MK, Vichos I, Walker L, Wand ME, Watkins B, Welch J, West AJ, White C, White R, Williams P, Woodyer M, Worth AT, Wright D, Wrin T, Yao XL, Zbarcea DA, Zizi D (2021). Safety and immunogenicity of ChAdOx1 nCoV-19 vaccine administered in a prime-boost regimen in young and old adults (COV002): a single-blind, randomised, controlled, phase 2/3 trial. Lancet..

[52] Weng NP (2006). Aging of the immune system: how much can the adaptive immune system adapt?. Immunity..

[53] Andrew MK, Shinde V, Ye L, Hatchette T, Haguinet F, Dos Santos G, McElhaney JE, Ambrose A, Boivin G, Bowie W, Chit A, ElSherif M, Green K, Halperin S, Ibarguchi B, Johnstone J, Katz K, Langley J, Leblanc J, Loeb M, MacKinnon-Cameron D, McCarthy A, McGeer A, Powis J, Richardson D, Semret M, Stiver G, Trottier S, Valiquette L, Webster D, McNeil SA, for the Serious Outcomes Surveillance Network of the Public Health Agency of Canada/Canadian Institutes of Health Research Influenza Research Network (PCIRN) and the Toronto Invasive Bacterial Diseases Network (TIBDN) (2017). The importance of frailty in the assessment of influenza vaccine effectiveness against influenza-related hospitalization in elderly people. J Infect Dis.

[54] Yao X, Hamilton RG, Weng NP, Xue QL, Bream JH, Li H, Tian J, Yeh SH, Resnick B, Xu X, Walston J, Fried LP, Leng SX (2011). Frailty is associated with impairment of vaccine-induced antibody response and increase in post-vaccination influenza infection in community-dwelling older adults. Vaccine..

[55] Torjesen I (2021). Covid-19: doctors in Norway told to assess severely frail patients for vaccination. BMJ..

[56] Torjesen I (2021). Covid-19: Norway investigates 23 deaths in frail elderly patients after vaccination. BMJ..

[57] Carly W. Age and frailty are independently associated with increased COVID-19 mortality and increased care needs in survivors: results of an international multi-centre study. Age Ageing. 2021;50(3):617–30. 10.1093/ageing/afab026 PMC7929433 33543243

[58] Osuafor CN, Davidson C, Mackett AJ, Goujon M, Van Der Poel L, Taylor V (2021). Clinical features, inpatient trajectories and frailty in older inpatients with COVID-19: a retrospective observational study. Geriatrics (Basel).

[59] Hewitt J, Carter B, Vilches-Moraga A, Quinn TJ, Braude P, Verduri A, Pearce L, Stechman M, Short R, Price A, Collins JT, Bruce E, Einarsson A, Rickard F, Mitchell E, Holloway M, Hesford J, Barlow-Pay F, Clini E, Myint PK, Moug SJ, McCarthy K, Davey C, Jones S, Lunstone K, Cavenagh A, Silver C, Telford T, Simmons R, Mutasem TEJ, Singh S, Paxton D, Harris W, Galbraith N, Bhatti E, Edwards J, Duffy S, Bisset C, Alexander R, Garcia M, Sangani S, Kneen T, Lee T, McGovern A, Guaraldi G (2020). The effect of frailty on survival in patients with COVID-19 (COPE): a multicentre, European, observational cohort study. Lancet Public Health.

[60] Aw D, Woodrow L, Ogliari G, Harwood R (2020). Association of frailty with mortality in older inpatients with Covid-19: a cohort study. Age Ageing.

[61] Labenz C, Kremer WM, Schattenberg JM, Worns MA, Toenges G, Weinmann A (2020). Clinical frailty scale for risk stratification in patients with SARS-CoV-2 infection. J Investig Med.

[62] Kow CS, Hasan SS, Thiruchelvam K, Aldeyab M (2021). Association of frailty and mortality in patients with COVID-19: a meta-analysis. Br J Anaesth.

[63] Darvall JN, Bellomo R, Bailey M, Paul E, Young PJ, Rockwood K, Pilcher D (2020). Frailty and outcomes from pneumonia in critical illness: a population-based cohort study. Br J Anaesth.

[64] Chinnadurai R, Ogedengbe O, Agarwal P, Money-Coomes S, Abdurrahman AZ, Mohammed S, Kalra PA, Rothwell N, Pradhan S (2020). Older age and frailty are the chief predictors of mortality in COVID-19 patients admitted to an acute medical unit in a secondary care setting- a cohort study. BMC Geriatr.

[65] Kompanje EJ, Piers RD, Benoit DD (2013). Causes and consequences of disproportionate care in intensive care medicine. Curr Opin Crit Care.

[66] Meyfroidt G, Vlieghe E, Biston P, De Decker K, Wittebole X, Collin V, et al. Ethical principles concerning proportionality of critical care during the COVID-19 pandemic: advice by the Belgian Society of IC medicine. 2020. Retrieved 2/04/2020, from: https://www.hartcentrumhasselt.be/professioneel/nieuws-professioneel/ethical-principles-concerning-proportionalityof-critical-care-during-the-covid-19-pandemic-advice-by-the-belgian-society-of-ic-medicine.

[67] ICNARC. ICNARC report on COVID-19 in critical care. 26 February 2021 [cited 2021 03 March]. Available from: https://www.icnarc.org/DataServices/Attachments/Download/93fb3700-7178-eb11-912e-00505601089b. [Accessed 3 Mar 2021].

[68] CDC. Morbidity and Mortality Weekly Report, Severe Outcomes Among Patients with Coronavirus Disease 2019 (COVID-19)—United States, February 12–March 16, 2020 2020 [Available from: https://www.cdc.gov/mmwr/volumes/69/wr/mm6912e2.htm. [Accessed 18 Apr 2020]. 10.15585/mmwr.mm6912e2 PMC7725513 32214079

[69] Richardson S, Hirsch JS, Narasimhan M, Crawford JM, McGinn T, Davidson KW, Barnaby DP, Becker LB, Chelico JD, Cohen SL, Cookingham J, Coppa K, Diefenbach MA, Dominello AJ, Duer-Hefele J, Falzon L, Gitlin J, Hajizadeh N, Harvin TG, Hirschwerk DA, Kim EJ, Kozel ZM, Marrast LM, Mogavero JN, Osorio GA, Qiu M, Zanos TP, and the Northwell COVID-19 Research Consortium (2020). Presenting characteristics, comorbidities, and outcomes among 5700 patients hospitalized with COVID-19 in the new York City area. JAMA..

[70] Lintern S. We are making difficult choices': Italian doctor tells of struggle against coronavirus: Independent; 2020 [Available from: https://www.independent.co.uk/news/health/coronavirus-italy-hospitals-doctor-lockdown-quarantine-intensive-care-a9401186.html. [Accessed 18 Apr 2020].

[71] Scheidegger D, Fumeaux T, Hurst S. COVID-19 pandemic: triage for intensive-care treatment under resource scarcity. Swiss Med Wkly. 2020;150. 10.4414/smw.2020.20229 32208495

[72] NYSDH. New York State Department of Health VENTILATOR ALLOCATION GUIDELINES: New York State Department of Health; 2015 [cited 2020 April]. Available from: https://www.health.ny.gov/regulations/task_force/reports_publications/docs/ventilator_guidelines.pdf. [Accessed 30 Apr 2020].

[73] Ministry of health, report on ethical issues in pandemic situations: SARS-CoV-2 Spain. 2020 [Available from: https://rm.coe.int/pandemic-covid-19-spain-eng/16809e3a78. [accessed 2020 June 02].

[74] The University of Pittsburgh, School of Medicine, Department of critical care. Allocation of Scarce Critical Care Resources During a Public Health Emergency: The University of Pittsburgh, School of Medicine; 2020 [updated 03.04.2020; cited 2020 April]. Available from: https://ccm.pitt.edu/sites/default/files/UnivPittsburgh_ModelHospitalResourcePolicy.pdf. [Accessed 10 Apr 2020].

[75] NICE. COVID-19 rapid guideline: critical care in adults UK: National Institute for Health and Care excellence; 2020 [cited 2021 March 10]. Available from: https://www.nice.org.uk/guidance/ng159/chapter/2-Admission-to-critical-care. [Accessed 10 Mar 2021]. 33497153

[76] Kunz R, Minder M (2020). COVID-19 pandemic: palliative care for elderly and frail patients at home and in residential and nursing homes. Swiss Med Wkly.

[77] Guralnik JM, LaCroix AZ, Branch LG, Kasl SV, Wallace RB (1991). Morbidity and disability in older persons in the years prior to death. Am J Public Health.

[78] Flaatten H, De Lange DW, Morandi A, Andersen FH, Artigas A, Bertolini G (2017). The impact of frailty on ICU and 30-day mortality and the level of care in very elderly patients (>/= 80 years). Intensive Care Med.

[79] Ferrante LE, Pisani MA, Murphy TE, Gahbauer EA, Leo-Summers LS, Gill TM (2018). The Association of Frailty with Post-ICU disability, nursing home admission, and mortality: a longitudinal study. Chest..

[80] Truog RD, Mitchell C, Daley GQ (2020). The toughest triage - allocating ventilators in a pandemic. N Engl J Med.

[81] Ensrud KE, Kats AM, Schousboe JT, Taylor BC, Cawthon PM, Hillier TA, Yaffe K, Cummings SR, Cauley JA, Langsetmo L, Study of Osteoporotic Fractures (2018). Frailty phenotype and healthcare costs and utilization in older women. J Am Geriatr Soc.

[82] Rajabali N, Rolfson D, Bagshaw SM (2016). Assessment and utility of frailty measures in critical illness, cardiology, and cardiac surgery. Can J Cardiol.

[83] Faller JW, Pereira DDN, de Souza S, Nampo FK, Orlandi FS, Matumoto S (2019). Instruments for the detection of frailty syndrome in older adults: a systematic review. PLoS One.

[84] Petermann-Rocha F, Hanlon P, Gray SR, Welsh P, Gill JM, Foster H (2020). Comparison of two different frailty measurements and risk of hospitalisation or death from COVID-19: findings from UK biobank. BMC Med.

[85] Fried LP, Tangen CM, Walston J, Newman AB, Hirsch C, Gottdiener J, Seeman T, Tracy R, Kop WJ, Burke G, McBurnie MA (2001). Frailty in older adults: evidence for a phenotype. J Gerontol A Biol Sci Med Sci.

[86] Rolfson DB, Majumdar SR, Tsuyuki RT, Tahir A, Rockwood K (2006). Validity and reliability of the Edmonton frail scale. Age Ageing.

[87] Steverink N (2001). Measuring frailty: developing and testing the GFI (Groningen frailty Indicator). The Gerontologist.

[88] Del Brutto OH, Costa AF, Recalde BY, Mera RM (2020). Frailty and SARS-CoV-2 infection. A population-based study in a highly endemic village. J Neurol Sci.

[89] Gilbert T, Neuburger J, Kraindler J, Keeble E, Smith P, Ariti C, Arora S, Street A, Parker S, Roberts HC, Bardsley M, Conroy S (2018). Development and validation of a hospital frailty risk score focusing on older people in acute care settings using electronic hospital records: an observational study. Lancet.

[90] Kundi H, Çetin EHÖ, Canpolat U, Aras S, Celik O, Ata N, Birinci S, Çay S, Özeke Ö, Tanboğa IH, Topaloğlu S (2020). The role of frailty on adverse outcomes among older patients with COVID-19. J Infect.

[91] Bruno RR, Wernly B, Flaatten H, Schölzel F, Kelm M, Jung C (2019). The hospital frailty risk score is of limited value in intensive care unit patients. Crit Care.

[92] Searle SD, Mitnitski A, Gahbauer EA, Gill TM, Rockwood K (2008). A standard procedure for creating a frailty index. BMC Geriatr.

[93] Bellelli G, Rebora P, Valsecchi MG, Bonfanti P, Citerio G, Members C-MT (2020). Frailty index predicts poor outcome in COVID-19 patients. Intensive Care Med.

[94] O'Caoimh R, Costello M, Small C, Spooner L, Flannery A, O'Reilly L (2019). Comparison of frailty screening instruments in the emergency department. Int J Environ Res Public Health.

[95] Raiche M, Hebert R, Dubois MF (2008). PRISMA-7: a case-finding tool to identify older adults with moderate to severe disabilities. Arch Gerontol Geriatr.

[96] Morley JE, Malmstrom TK, Miller DK (2012). A simple frailty questionnaire (FRAIL) predicts outcomes in middle aged African Americans. J Nutr Health Aging.

[97] Rockwood K, Song X, MacKnight C, Bergman H, Hogan DB, McDowell I, Mitnitski A (2005). A global clinical measure of fitness and frailty in elderly people. CMAJ..

[98] Liang YD, Zhang YN, Li YM, Chen YH, Xu JY, Liu M, Li J, Ma Z, Qiao LL, Wang Z, Yang JF, Wang H (2019). Identification of frailty and its risk factors in elderly hospitalized patients from different wards: a cross-sectional study in China. Clin Interv Aging.

[99] Pugh RJ, Ellison A, Pye K, Subbe CP, Thorpe CM, Lone NI, et al. Feasibility and reliability of frailty assessment in the critically ill: a systematic review. Critical care. 2018;22(1):49. DOI: 10.1186/s13054-018-1953-9. 10.1186/s13054-018-1953-9 PMC6389132 29478414

[100] Wallis SJ, Wall J, Biram RW, Romero-Ortuno R (2015). Association of the clinical frailty scale with hospital outcomes. QJM..

[101] Guidet B, de Lange DW, Boumendil A, Leaver S, Watson X, Boulanger C (2020). The contribution of frailty, cognition, activity of daily life and comorbidities on outcome in acutely admitted patients over 80 years in European ICUs: the VIP2 study. Intensive Care Med.

[102] Gregorevic KJ, Hubbard RE, Lim WK, Katz B (2016). The clinical frailty scale predicts functional decline and mortality when used by junior medical staff: a prospective cohort study. BMC Geriatr.

[103] Church S, Rogers E, Rockwood K, Theou O (2020). A scoping review of the clinical frailty scale. BMC Geriatr.

[104] Dent E, Morley JE, Cruz-Jentoft AJ, Woodhouse L, Rodriguez-Manas L, Fried LP (2019). Physical frailty: ICFSR international clinical practice guidelines for identification and management. J Nutr Health Aging.

[105] Blomaard LC, van der Linden CMJ, van der Bol JM, Jansen SWM, Polinder-Bos HA, Willems HC, Festen J, Barten DG, Borgers AJ, Bos JC, van den Bos F, de Brouwer EJM, van Deudekom FJA, van Dijk SC, Emmelot-Vonk MH, Geels RES, van de Glind EMM, de Groot B, Hempenius L, Kamper AM, Kampschreur LM, de Koning MMM, Labots G, Looman R, Lucke JA, Maas HAAM, Mattace-Raso FUS, el Moussaoui R, van Munster BC, van Nieuwkoop C, Oosterwijk L(BLE), Regtuijt M(EM), Robben SHM, Ruiter R, Salarbaks AM, Schouten HJ, Smit OM, Smits RAL, Spies PE, Vreeswijk R, de Vries OJ, Wijngaarden MA, Wyers CE, Mooijaart SP (2021). Frailty is associated with in-hospital mortality in older hospitalised COVID-19 patients in the Netherlands: the COVID-OLD study. Age Ageing.

[106] De Smet R, Mellaerts B, Vandewinckele H, Lybeert P, Frans E, Ombelet S (2020). Frailty and mortality in hospitalized older adults with COVID-19: retrospective observational study. J Am Med Dir Assoc.

[107] Owen RK, Conroy SP, Taub N, Jones W, Bryden D, Pareek M, Faull C, Abrams KR, Davis D, Banerjee J (2021). Comparing associations between frailty and mortality in hospitalised older adults with or without COVID-19 infection: a retrospective observational study using electronic health records. Age Ageing.

[108] Miles A, Webb TE, McLoughlin BC, Mannan I, Rather A, Knopp P (2020). Outcomes from COVID-19 across the range of frailty: excess mortality in fitter older people. Eur Geriatric Med.

[109] Gilis M, Chagrot N, Koeberle S, Tannou T, Brunel AS, Chirouze C, Bouiller K (2021). Older adults with SARS-CoV-2 infection: utility of the clinical frailty scale to predict mortality. J Med Virol.

[110] Sablerolles RS, Lafeber M, van Kempen JA, van de Loo BP, Boersma E, Rietdijk WJ (2021). Association between clinical frailty scale score and hospital mortality in adult patients with COVID-19 (COMET): an international, multicentre, retrospective, observational cohort study. Lancet Healthy Longevity.

