# Supplementary material for: Different aspects of frailty and COVID-19: points to consider in the current pandemic and future ones
Source: BMC Geriatr. 2021 Jun 27;21:389. doi: 10.1186/s12877-021-02316-5 (PMC8236311; doi:10.1186/s12877-021-02316-5)
Supplement: Supplementary file 1 — Additional file 1: Supplementary Table 1. The main frailty screening tools used in clinical practice. [file 12877_2021_2316_MOESM1_ESM.docx]

**Supplementary Table 1 – The main frailty screening tools used in clinical practice**

| **Instrument** | **Components** | **Scoring** |
| --- | --- | --- |
| Frailty Phenotype (FP) | - Composed of five components 1. weakness, 2.slowness, 3.unintentional weight loss, 4.exhaustion, and 5.low physical activity - Every component has one point if it is answered with Yes. | - 0 points = Robust - 1-2 points= pre-frail - 3-5= frail |
| Frailty Index (FI) | - It calculates accumulated deficits.   - Original FI is 38 deficits   - Self-reported FI is 36 deficits   - Laboratory FI is 32 deficits - Calculated as a ratio of deficits present to the total number of deficits considered | - The ratio is interpreted as:   - Excellent = 0   - Very Good = 0.25   - Good = 0.5   - Fair = 0.75   - Poor = 1 |
| Clinical Frailty Scale (CFS) | Clinical judgment, ranging from very fit to severely frail: 1 = Very fit; 2 = Well; 3 = Well, with treated comorbid disease; 4 = Apparently vulnerable; 5 = Mildly frail; 6 = Moderately frail; 7 = Severely frail (or terminally ill) | - The physician assigns a score of 1 to 7 based on clinical judgment. - Physicians are making the initial assessment given access to diagnoses and assessments related to these variables and other measures of comorbidity, function and associated features that inform clinical judgments about the severity of frailty. - A multidisciplinary team performs a secondary review and scoring. |
| PRISMA-7 Questionnaire | Seven yes or no self-reported questions about 1) Age; 2) Sex; 3) Health problems that require a limit on activities; 4) Help needed from someone regularly; 5) Health problems that require one to stay at home; 6) Having someone to count on if needed, and 7) Regular use of an assistive device for walking. | If there is yes to three or more of the seven questions = potential frailty |
| Groningen frailty indicator (GFI) | - A self-reporting tool. - The GFI is a 15-item tool. - The maximum score of 15. | - ≥ Four is considered frail. |
| FRAIL Scale | - A self-reporting test that assesses an older adult in terms of Fatigue, Resistance, Ambulation, Illnesses and Loss of Weight - Every component has one point if it is answered with Yes. | - 0 points = Robust - 1-2 points= pre-frail - 3-5 points = frail |
| Edmonton Frailty Scale (EFS) | - Self-reporting - It consists of eleven questions. - It covers cognitive function, general health status, functional independence, social support, medications, nutrition state, mood, incontinence, and balance/mobility. | - A point-score with a maximum score of 17, - Individuals being categorized into robust (0–4 ), pre-frail (5–6 ) and frail (≥7 points). |
| The Hospital Frailty Risk Score (HFRS) | - ICD-10-code-based algorithm - 109 ICD-10 codes - Diagnosis- Points Awarded | Low risk (<5), Intermediate risk (5-15) High risk (>15) |

*ICD = International Statistical Classification of Diseases and Related Health Problems, PRISMA= Program of Research on Integration of Services for the Maintenance of Autonomy.*
