# Supplementary material for: Different aspects of frailty and COVID-19: points to consider in the current pandemic and future ones
Source: BMC Geriatr. 2021 Jun 27;21:389. doi: 10.1186/s12877-021-02316-5 (PMC8236311; doi:10.1186/s12877-021-02316-5)
Supplement: Supplementary file 2 — Additional file 2: Table 2. Frailty assessment tools that were used during the COVOD-19 pandemic. [file 12877_2021_2316_MOESM2_ESM.docx]

| **Frailty assessment tool** | **Type** | **Used for patients diagnosed with COVID-19** |
| --- | --- | --- |
| CFS | Clinician judgment | Yes |
| EFS | Calculation | Yes |
| FRAIL Scale | Self-reporting | Yes |
| FI | It is generated from medical records and laboratory parameters. | Yes |
| HFRS | It is generated from medical records | Yes |

**Table 2. Frailty assessment tools that were used during the COVOD-19 pandemic**

*Clinical frailty scale (CFS), Edmonton Frail Scale (EFS), Frailty Index (FI), Hospital Frailty Risk Score ( HFRS)*
